# COVID-19: Uncertainties from Conception to Birth

**DOI:** 10.1055/s-0040-1721856

**Published:** 2021-01-29

**Authors:** Bruno Ramalho de Carvalho, Karina de Sá Adami, Walusa Assad Gonçalves-Ferri, Marise Samama, Rui Alberto Ferriani, Alessandra Cristina Marcolin

**Affiliations:** 1 Hospital Sírio-Libanês, Brasília, DF, Brazil; 2 Maternidade Climério de Oliveira, Universidade Federal da Bahia, Salvador, BA, Brazil; 3 Department of Pediatrics, Faculdade de Medicina de Ribeirão Preto, Universidade de São Paulo, Ribeirão Preto, SP, Brazil; 4 Department of Gynecology, Escola Paulista de Medicina, Universidade Federal de São Paulo, SP, Brazil; 5 Department of Gynecology and Obstetrics, Faculdade de Medicina de Ribeirão Preto, Universidade de São Paulo, Ribeirão Preto, SP, Brazil

**Keywords:** COVID-19, severe acute respiratory syndrome coronavirus 2, ovary, testis, conception, pregnancy, Covid-19, síndrome respiratória aguda grave pelo coronavírus 2, ovário, testículo, concepção, gravidez

## Abstract

Scientific information on the impact of the new coronavirus (SARS-CoV-2) on the health of pregnant women, fetuses and newborns is considered of limited confidence, lacking good-quality evidence, and drawing biased conclusions. As a matter of fact, the initial impressions that the evolution of COVID-19 was no different between pregnant and non-pregnant women, and that SARS-CoV-2 was not vertically transmitted, are confronted by the documentation of worsening of the disease during pregnancy, poor obstetric outcomes, and the possibility of vertical transmission. The present article aims to compile the data available on the association of COVID-19 and reproductive events, from conception to birth.

## Resumo

As informações científicas sobre o impacto do novo coronavírus, SARS-CoV-2, na saúde de gestantes, fetos e recém-nascidos são consideradas de confiabilidade limitada, sem evidências de boa qualidade, e levam a conclusões enviesadas. De fato, as impressões iniciais de que a evolução da Covid-19 não era diferente entre mulheres grávidas e não grávidas, e de que o SARS-CoV-2 não era transmitido verticalmente, são confrontadas pela documentação de agravamentos da doença durante a gravidez, resultados obstétricos negativos, e a possibilidade de transmissão vertical. Este artigo tem como objetivo compilar os dados disponíveis sobre a associação entre a Covid-19 e os eventos reprodutivos, desde a concepção até o nascimento.

## Introduction

In the last 8 months, the world has been experiencing a pandemic caused by the new coronavirus (SARS-CoV-2). Starting with the first reported cases of pneumonia in the city of Wuhan, Hubei province, China, 1 by August 16, 2020, 21,294,845 COVID-19 cases had already been confirmed, with a total of 761,779 deaths. 2 On that date, the Brazilian Ministry of Health had registered 3,340,197 cases of the disease, and 107,852 deaths. 3

It is a fact that scientific information on the impact of SARS-CoV-2 on the health of pregnant women, fetuses and newborns is considered of limited confidence, lacking good-quality evidence, and drawing biased conclusions. As a matter of fact, the initial impressions that the evolution of COVID-19 was no different between pregnant and non-pregnant women, 4 5 6 and that SARS-CoV-2 was not vertically transmitted, 4 7 8 are confronted by the documentation of worsening of the disease during pregnancy, poor obstetric outcomes, 9 10 11 12 13 14 and the possibility of vertical transmission. 15 16 17 18 19 20

Of note, uncertainty is the rule at the moment. Some state that, then, there are no convincing reasons to recommend people to avoid pregnancy. 21 In contrast, it seems that there are at least 5 uncertainties/reasons to advise against pregnancy at this time, if it is possible, namely: 1) there is confounding evidence on the evolution of COVID-19 in pregnant and non-pregnant women; 2) despite the fact that the bulk of the literature suggests the effectiveness of the placental barrier in preventing fetal infection by SARS-CoV-2, fewreports 15 20 indicating vertical transmission sustain medical concerns; 3) it is not clearly known whether infection by SARS-CoV-2 in the first trimester can cause birth defects or pregnancy loss; 4) it is not known whether the newborns of women infected by SARS-CoV-2 will have the same clinical evolution as those born to healthy mothers; and 5) the possibility of SARS-CoV-2 transmission through the sexual route, and the role of its theoretical effect on the gonads and gametes, in vivo and in vitro.

The present review article aims to compile available data on each of the aforementioned uncertainties, providing theoretical background for counseling and caring for women who are planning a pregnancy, those who are pregnant, or those who have given birth during the COVID-19 pandemic.

## SARS-CoV-2 in Reproductive Tissues and Gametes

The expression of angiotensin-converting enzyme 2 (ACE2) and other viral entry facilitators in male and female gonads and gametes, as well as the endometrium, 22 23 24 25 raises concerns on the interference of SARS-CoV-2 on reproductive health and conception. 26 To date, it is not known if the new coronavirus affects reproductive function via the ACE2 receptors, and which consequences, if any, the infection by the virus would have on the quality of the gametes, embryo development and implantation, or pregnancy in its very beginning. 27

Indeed, there are reports of human orchitis related to other coronaviruses. 28 Then, the possibility of immediate or long-term SARS-CoV-2-related impairment in the male reproductive functions is a genuine concern. Theoretically, inflammation in the seminiferous epithelium, Leydig and Sertoli cells, and the epididymis could lead to oxidative stress and sperm DNA fragmentation, as well as diminished testosterone secretion and disrupted spermatogenesis. 29

Regarding conception, the possibility of sexual transmission is another matter of concern. SARS-CoV-2 has already been detected in the small intestine, liver, pancreas, kidney and sweat glands, so, at least theoretically, there are other potential routes of transmission than the respiratory one. 30 The data available on the sexual route of transmission are controversial. In a Chinese cohort study, 31 SARS-CoV-2 was detected in semen samples from infected patients, both in the acute and in the recovery phases. In contrast, other reports did not find the virus in semen collected 8 days after COVID-19 diagnosis, 32 or 30 days. 33 Divergent findings about SARS-CoV-2 in the semen support the need for larger studies, which should not just confirm the presence of the virus in the semen, but provide information about its length of stay and the possibility of transmission by sexual contact.

## COVID-19 during Pregnancy

Maternal and fetal complications were documented in previous epidemics caused by other coronaviruses, namely the severe acute respiratory syndrome (SARS) 34 and the Middle East respiratory syndrome (MERS). 35 In addition to historical aspects, physiological adaptations during pregnancy are usually considered to be potential factors of vulnerability to any type of infection. Moreover, some authors suggest that pregnant women are more vulnerable to infective respiratory agents than the general population, and they may respond to COVID-19 with a “cytokine storm,” which may lead to severe morbidity. 36 Beyond this, the signs or symptoms related to pregnancy may overlap with other symptoms of COVID-19, thereby making the diagnosis challenging. So, as a precaution, pregnancy and the puerperium are now considered high-risk situations for severe illness from COVID-19. 37 38

In June 2020, a systematic review 39 of 755 pregnant women presenting with COVID-19 provided only poor-quality evidence, and the authors could not rule out potential worsening of the clinical conditions of pregnant women infected with SARS-CoV-2, or whether the infection is associated with comorbidities or not. Actually, the absence of studies with reliable methodology still greatly limits data interpretation. Thus, it cannot yet be said that the disease evolves in the same way in pregnant and non-pregnant women, nor can pregnancy be excluded from the list of potential risk conditions.

In the United States, the Centers for Disease Control and Prevention (CDC) has recently published a series 12 of 8,207 pregnant women in the United States, associating pregnancy to an increased risk of hospitalization (31.5% in pregnant women *versus* 5.8% in non-pregnant women; adjusted relative risk [aRR] = 5.4; 95% confidence interval [95%CI] = 5.1 to 5.6), admission to the Intensive Care Unit (aRR = 1.5; 95%CI = 1.2 to 1.8), and the need for mechanical ventilation (aRR = 1.7; 95%CI = 1.2 to 2.4) due to COVID-19. Nevertheless, the increase in morbidity was not reflected in increased mortality, which was similar between pregnant and non-pregnant women (0.2% in both groups; aRR = 0.9; 95%CI = 0.5 to 1.5).

Recent data show that most of the women (∼ 85%) contracting SARS-CoV-2 will exhibit mild characteristics of the disease. The rates of severe disease vary between 9.3% and 11.1%, and the rates of critical disease vary between 2% and 6.9%, which are just as similar to the rates for the general population. 40 However, local scenarios of care for pregnant women should also be taken into account to better advise the women about the possibility of becoming pregnant. In Brazil, for example, a country with high rates of maternal mortality when compared with developed countries, 124 pregnant or postpartum women have died due to COVID-19 until June 18, 2020–representing a mortality rate of 12.7% –, a figure that currently surpasses the total number of COVID-19-related maternal deaths reported throughout the rest of the world. These data suggest an aggravation of the bad preexisting conditions of prenatal care due to COVID-19 infection. 10

In the context of the COVID-19 pandemic, the high rates of preterm birth, cesarean section, 41 42 and preeclampsia, 43 and the increased risk of admissions to the intensive care unit 12 persist as the most common reasons for concern. Outstandingly, the most frequent COVID-19 adverse obstetric outcome seems to be preterm birth, occurring in 41.1% (95%CI: 25.6 to 57.6) of cases. 42 In view of this little-known disease, it is recommended to consider pregnancy a potential aggravating factor for COVID-19, as is customary for other diseases, and, conversely, to consider this infection a cause of negative obstetric and perinatal outcomes. As a precautionary action, it is reasonable to institute unrestricted monitoring measures for pregnant women infected with SARS-CoV-2, and even to suggest that they should be rigorously tested before delivery or before the first contact with the newborns. 39

## Evidence of Vertical Transmission of SARS-CoV-2

Vertical transmission of previous coronaviruses was hypothesized in preliminary studies, 44 45 but such an occurrence was not reported with either SARS or MERS; therefore, it seems that the likelihood of intrauterine maternal-fetal transmission of coronaviruses is low. 46 47

To date, it cannot be said that the placental barrier is capable of preventing the vertical transmission of SARS-CoV-2. Often times, tests of the amniotic fluid, cord blood, and neonatal throat swab samples were not able to evidence the vertical transmission of SARS-CoV-2, at least in mid- 8 and late pregnancy. 42 47 However, third trimester placentas from COVID-19 patients have been proven to be more likely to present maternal/fetal vascular malperfusion, 48 and the existence of vertical transmission of SARS-CoV-2 has already been demonstrated in some case reports 49 50 and small case series. 51 52 Moreover, the detection of anti-SARS-CoV-2 immunoglobulin M (IgM) in cord and/or early-life neonatal blood supports the suspicion of intrauterine infection, since IgM is not transplacentally transferred from the mother to the fetus. 17

Recently, in early July 2020, during one of the virtual sessions of the Annual Meeting of the European Society of Human Reproduction and Embryology (ESHRE), researcher Wafaa Essahib presented evidence of the expression of ACE2 and other viral-entry facilitators in human oocytes and blastocysts, supporting a new hypothesis of transmission of the virus from the mother to the embryo, which would be cause for apprehension for initial pregnancies under COVID-19. 53 Moreover, the expression of the ACE2 receptor has been reported in the placenta, which may increase the risk of vertical transmission of the virus. 54

It is worth saying that, if maternal-fetal transmission happens, little is known about its impact over embryogenesis, morphogenesis, fetal development, and health.

## The Health of the Fetus and the Newborn

Initial data suggested that maternal SARS-CoV-2 infection does not seem to be more harmful in the first trimester than it is throughout the whole pregnancy. Infection was not associated with increased thickness of the nuchal translucency or risk of pregnancy loss. 55 However, other viruses, depending on the gestational age at the moment of infection, have been associated with congenital anomalies and developmental delay later on, like Zika. 56 Thus, the little time since the first studies on the behavior of SARS-CoV-2 is insufficient to evaluate its real impact on neonates.

The RNA of SARS-CoV-2 has already been found in placental and amniotic membrane samples, indicating the possibility of fetal viral exposure during labor and delivery. 57 Placental infection has also been demonstrated as one of the negative outcomes of pregnancy, not just by identification of viral RNA, but by histological findings of subchorial inflammatory infiltrates, increased intervillous fibrin deposition, and funisitis (suggesting inflammatory response from the fetus). 14

According to the systematic review of 598 newborns, strong evidence of SARS-CoV-2 infection was not available until June 2020, especially considering the lack of reliable information on the care provided during and after delivery, as well as the lack of adequate collection of biological samples from newborns for the SARS-CoV-2 test. 39 Indeed, the data available to date suggest that COVID-19 infection is uncommon among newborns, which are frequently asymptomatic. Furthermore, it was not possible to associate a higher risk of infection to vaginal birth, breastfeeding or close contact with the infected mother. 58

It is worth highlighting the fact that there has been a decrease in extremely premature births during the COVID-19 pandemic. 59 60 There are still no definite explanations for this unprecedent reduction, but there are two hypotheses: the disease leads to a greater number of early pregnancy losses; or the measures of social distancing and confinement lead to lower exposure of pregnant women to the behavioral and socio-environmental factors linked to prematurity.

## Postponing Pregnancy versus Age-related Fertility Loss

Since the deleterious effect of postponing pregnancy by a few months may not be significant, 61 62 63 64 65 even for women of advanced reproductive age or with diminished ovarian reserve, 64 such a decision has been encouraged by some authors. 65 66

In a very recent retrospective cohort study on in vitro fertilization treatment for women with diminished ovarian reserves, Romanski et al. 65 observed that a delay of up to 180 days did not seem to negatively affect live birth rates, even for women with anti-Mullerian hormone levels < 0.5 ng/mL, or those older than 40 years of age. The authors recognized a potential selection bias in their study, but considered the results sufficient to reassure that short-term delay in the treatment will not affect its outcomes, in situations that such a delay is deemed necessary or prudent, like the current COVID-19 pandemic.

For cases of infertility that require assisted reproduction, the dilemmas can be even greater. Advice on whether or not to postpone treatment includes information on the lack of knowledge of the effects of COVID-19 on pregnancy, as well as the possible risks of vertical transmissibility and transmission via the gametes, as well as risks of the virus being stored in the gametes and the embryo. 26 66 It should be emphasized that the treatment per se lasts several days, and requires several return visits, and, depending on the region's epidemiological situation, it may be inadvisable for patients to move and expose themselves to medical environments. Besides that, the infection can occur during the treatment period, causing suspension at any stage, which can generate significant emotional distress. Finally, the lack of consensus on screening methods contributes to the uncertainty, since the diagnostic tests contain highly-variable sensitivities and specificities, and unfortunately there is no consensus on which one is the best and safest to detect the disease in real time. 67

## Perspectives for the Future

### Pregnancy and Delivery

At this moment, the best knowledge on COVID-19 in pregnancy is based on observational studies, case series and reports, as well as estimates from the surveillance of sanitary authorities in each country. These provide insufficient information to consider changes in the management and definition of the appropriate interventions. Wise strategies minimizing the risk for the pregnancy and poor obstetric outcomes, mainly those associated to preterm birth, preeclampsia, and cesarean section, will probably be defined once there is wide knowledge of the disease, which will come with time and experience.

There is no doubt that many aspects remain unclear to date, and randomized clinical trials are expected to provide knowledge from preconception counseling and preventive measures during prenatal care to clinical presentation and management of the infection during pregnancy and the puerperium. Oxford-Horrey et al. 68 opportunely highlight that supportive care must focus on recovery rather than delivery as the main treatment endpoint. Unfortunately, the inclusion of pregnant women in registered trials still seems to be small: among all 3,009 studies registered for COVID-19 in the ClinicalTrials.gov database ( https://clinicaltrials.gov ) on August 16, 2020, only 66 (∼ 2.2%) included pregnant women, of which only 6 had been completed, 1 had been withdrawn, and 20 were not yet recruiting.

Specifically in Brazil, the high rates of maternal deaths related to COVID-19 might be associated with poor quality in prenatal and obstetric care. Indeed, SARS-CoV-2 may be an aggravating agent of a previous inflammatory status present in hypertensive disease and/or obesity, which are very prevalent in the country. However, social risks and limited access to health care are probably associated to late proper care, potentially contributing to maternal deaths. 69

Finally, childbirth care under COVID-19 is still one of the main concerning aspects. However, it is worth observing that, to date, there is no evidence of the intrapartum transmission of SARS-CoV-2 to the newborn, and vaginal birth with appropriate precaution measures remains the mode of delivery in mild maternal disease. Moreover, it is essential to reinforce that interventions to shorten the lenght of the second stage of labor and/or to avoid delayed umbilical cord clamping and/or skin-to-skin contact are not supported by good evidence, as well as COVID-19 alone is not an indication for cesarean section. Instead, high rates of cesarean deliveries under COVID-19 are concerning, since they have the potential to promote clinical maternal deterioration. 70

### Fetal and/or Newborn Health

The effects of viremia on the embryo and/or the fetus in the first and second trimesters of pregnancy are poorly known. Actually, women who became pregnant just before or just after the beginning of the pandemic have not yet delivered their babies; therefore, information from the main pregnancy outcomes is still lacking. With regard to neonates, perhaps the great prospect for the future is the clarification of the causes of prematurity. To date, no one can determine if the higher rates of preterm birth under COVID-19 are spontaneous or elective due to maternal clinical complications or fetal compromise. As a matter of fact, it is not known whether children born to mothers infected by the SARS-CoV-2 will have the same clinical evolution as those born to healthy mothers. And such an answer is totally dependent on time.

### Availability and Reliability of Diagnostic Tests

COVID-19 screening in early pregnancy has been proposed as strategy to better plan maternal-fetal health surveillance programs in epidemic areas, combining serological tests and nucleic acid testing, 71 mainly for those women presenting for delivery. Those who defend universal screening for parturient women do so based on the benefits of determining hospital isolation practices and bed assignments, informing the neonatal care team, and guiding the use of personal protective equipment. 72 In contrast, the observation of 3,963 women admitted to labor and delivery in California, United States, resulted in a very low prevalence of positive SARS-CoV-2 testing, 73 raising doubts about the benefit of mass testing, especially in countries like Brazil, in which the testing of large populations still does not seem to be a reality.

### Vaccination

There is no doubt that scientific community is watching a race to launch a vaccine against SRAS-CoV-2. There is also no doubt that, with the first vaccine approved in a phase-III study, we will see one of the fastest, if not the fastest, launches of a vaccine in the entire history of active immunization. But there are questions that will be answered only over time: how effective will the vaccine be? Will there be enough distribution to interrupt or, at least, slow the spread of the virus? How safe will vaccinated women be to decide to become pregnant with the virus still spreading? And, finally, since the immune responses to vaccination in pregnant women may not be the same as those observed in non-pregnant women, 74 how will the safety of vaccination in pregnancy will be assessed?

## Conclusion

Health professionals and society as a whole have doubts about the impact of COVID-19 on reproductive outcomes, from the impact of SARS-CoV-2 in the gonads and/or gametes to its consequences to the newborn. Data regarding COVID-19 during pregnancy, as it happens with any other pathological condition, are uncertain, possibly due to missingness of pregnancy status, underreporting of cases and disease outcomes, inadequate collection of biological samples for testing, insufficient accuracy of the tests available, and inaccuracy of the mathematical models in predicting the number of asymptomatic individuals. Good quality data are urgently needed to support an adequate counseling to women attempting pregnancy and to those who are already pregnant. With limited evidence available to date, concerning uncertainties from conception to birth inevitably raise the possibility of postponing pregnancy, if possible, to a post-Covid-19 scenario or the one with an effective and broadly-distributed vaccine.
